# Refrigeration Capacity and Effect of Ageing on the Operation of Cellulose Evaporative Cooling Pads, by Wind Tunnel Analysis

**DOI:** 10.3390/ijerph16234690

**Published:** 2019-11-25

**Authors:** Antonio Franco-Salas, Araceli Peña-Fernández, Diego Luis Valera-Martínez

**Affiliations:** 1 ETSIA, University of Sevilla, Ctra. de Utrera km, 1, 41013 Sevilla, Spain; afranco@us.es; 2 CIAIMBITAL Research Centre, University of Almería, Ctra. de Sacramento s/n, 04120 Almería, Spain; apfernan@ual.es

**Keywords:** greenhouse, evaporative cooling pad, refrigeration capacity, loss of effectiveness

## Abstract

This study investigates the temperature reduction capacity and water consumption of a fan-pad system installed in a greenhouse located in the coastal regions of Almería. The suitability of this system for coastal zones with high environmental humidity during the summer is analyzed. Historical temperature and relative humidity series are studied, obtaining the thermal difference and maximum, medium, and minimum monthly water consumption of the pads based on the operation data of the pads. Despite the high relative humidity of the air in the hottest hours of the day, a decrease of 5.92 °C in the mean temperature and a water consumption of 13.55 l/h per square meter of an evaporative cooling pad are obtained in the month of August. Additionally, the operation of a cellulose evaporative cooling pad installed for 3 years in a greenhouse is analyzed in a wind tunnel and compared with that of a new pad of the same model. Over time and with low maintenance, the porosity of the pad decreases due to salt incrustation. The salt incrustation makes airflow more difficult in the pad, increasing the pressure drop by 170.04%; however, the air saturation efficiency of the pad increases by 6.6% due to the greater contact time between the air and the water.

## 1. Introduction

Evaporative cooling for crop protection is an interesting strategy for decreasing the entry temperature of the air in a greenhouse and maintaining adequate moisture content for plant production [1].

The greenhouses of Almeria (more than 31,000 ha) are based on family farming, providing a decent income to more than 15,000 families. They are a unique example in the world of wealth sharing and have been the engine of development of this entire region of southern Spain. The annual production of fruit and vegetables in the area exceeds 3.3 Mt, representing a final agricultural production of around EUR 2000 million. In addition, around 80% of production is destined for export. However, the area is subject to high temperatures that, aggravated by climate change, increase year after year. This makes it necessary to use greenhouse cooling systems based on water evaporation (i.e., cellulose pads-fan). Hence the extreme importance of this work to help the sustainability of intensive agriculture in the area, based on a very large number of small farms, where respect for the environment is extreme; for example, all greenhouses use biological control and much of the area is certified as organic production.

In addition, evaporative cooling produces substantial improvements in the climate control of greenhouses [2] and of intensive livestock operations [3,4]. With respect to traditional cooling systems, such as natural ventilation and shading, this method presents higher costs for both energy and water. These higher costs are compensated by the increase in quality, production, and crop yields. Evaporative cooling allows cultivation during the summer season, lengthening the campaign, or starting planting earlier, as well as by modifying the periods of maximum production. In the Mediterranean greenhouses on the Almería coast, fog nozzle systems combined with natural ventilation (fog systems) and systems of evaporative cooling pads combined with extractor fans (pad-fan systems) have both been used [5].

Both systems are more effective in reducing air temperature in regions characterized by hot and dry summers [6], but the suitability of these systems is compromised in hot and humid climates and coastal zones with high relative humidity.

Comparing these two evaporative cooling techniques, the pad-fan systems maintain more favorable and stable indoor conditions over time than those of fog nozzle systems [7]. Pad-fan systems also have a higher saturation efficiency [8], obtaining better results if combined with shading screens [9], in addition to consuming less electrical energy and water [10]. Nevertheless, pad-fan systems cause more microclimatic heterogeneity in the greenhouse, resulting in horizontal and vertical gradients of humidity, temperature, and CO_2_ concentration, which is not beneficial for the crop. On the other hand, pad-fan systems present a very interesting advantage for growers in warm and windy areas (such as south-eastern Spain); on windy days, the vents must be closed to protect the greenhouse structure and, in these conditions, the humid air mixture inside the greenhouse quickly becomes saturated with water vapor, losing the effectiveness of the nebulization. However, this circumstance does not affect greenhouses equipped with pad-fan systems.

The high temperatures and relative humidity during the summer months adversely affect crop production [11]. Another study indicates that refrigeration technologies are not sufficient to create the necessary conditions for crops in greenhouses in tropical and subtropical regions [12]. However, in interior regions where humidity is lower, greenhouses equipped with evaporative cooling pads and fans can reduce the temperature inside the greenhouse compared with that outside by between 4 and 6 °C. This reduction in temperature can be greater, between 4 and 12 °C, if combined with shading systems. Studies in greenhouses located in tropical and subtropical coastal zones with high environmental humidity analyzed the effect of shading screens combined with pad and fan systems. This combination of systems substantially reduces the interior temperature of a greenhouse, between 5 and 8 °C, compared with those of greenhouses without shade screens in the region of New Delhi (India) during the month of May [13], and in the case of Shanghai (China), the temperature was reduced by between 2 and 3 °C below the outside temperature with outside air conditions of 80% relative humidity [14].

One proposal to reduce the effect of excess environmental humidity is to use dehumidifiers or desiccant materials in combination with a cooling system that uses evaporative cooling pads and fans in greenhouses located in hot and humid climates [15]. The dehumidifier installed before the evaporative cooling pad reduces the moisture of the air and therefore the wet-bulb temperature, achieving a greater thermal difference. Compared with a greenhouse with evaporative cooling pads but without an air dehumidifier, the temperatures are decreased between 5.5 and 7.5 °C during the summer, prolonging the optimal season for the cultivation of lettuce to 3 to 6 months of the year and that of tomato and cucumber to between 7 and 12 months [16]. Similar results were obtained in an evaporative cooling system with a liquid desiccant, which reduced the mean daily maximum temperatures in a greenhouse by approximately 6 °C in relation to a conventional evaporative cooling system [17].

The Mediterranean climate is a variant of subtropical weather or of a temperate climate that is generally characterized by temperate winters and dry, hot summers. However, more specifically, there is the continental Mediterranean climate in the interior of Spain, which is characterized by continental temperatures with cold winters and very hot and dry summers, and the Mediterranean coastal climate, located from the border with Portugal to the border with France except for the arid climate domain of the south-east, which is characterized by mild winters and summers. The climate of the southeast of the peninsula is known as the Mediterranean sub-desert climate, which includes the coast of Almería and is characterized by low rainfall, high temperatures, and high environmental humidity in coastal regions.

Since the province of Almería (Spain) is the largest area of greenhouses in the world [18], the use of evaporative cooling systems in this region is compromised by high humidity, and thus, determining the decrease in the temperature and the consumption of water from evaporative cooling pads in greenhouses in coastal regions such as Almería is of interest. Both parameters can be achieved using a historical climatic data series representative of the region and using operating parameters for evaporative cooling pads obtained in a wind tunnel [19].

However, while there are many studies that analyze the dependence of different operating parameters of evaporative cooling pads, such as the saturation efficiency and the decrease in pressure with air flow, on the speed of the air flow and the pad thickness [20,21,22,23], few studies are focused on studying how these operating parameters vary over time with the deterioration or ageing of the evaporative cooling pads. Therefore, this study aims to analyze, firstly, the refrigeration capacity of a cellulose evaporation panel used in greenhouses located near the coast of Almeria (Spain), and secondly, to test the effect of the age of the evaporation panel on its operating parameters.

## 2. Materials and Methods

### 2.1. Procedure for the Evaluation of the Evaporative Cooling Pad Cooling Capacity

To determine the viability of fan-pad systems in greenhouses close to coastal regions, a climate data series from the last 11 years (2008–2018) was analyzed. The meteorological station used is located at an altitude of 10 m in the northern part of a multi-tunnel greenhouse located on the experimental farm belonging to the University of Almería in south-eastern Spain (36°51′ N, 2°16′ W, and an elevation of 87 m). The climatic data recorded outside the greenhouse for this study were the temperature, relative humidity, and solar radiation at intervals of 10 min. The weather station includes a BUTRON II box (Hortimax SL, Almería, Spain) with a Pt100 temperature sensor and a capacitive humidity sensor. The temperature measurement range is −25 °C to 75 °C with an accuracy of ± 0.01 °C, and the moisture measurement range is 0% to 100% with an accuracy of ±3%. The solar radiation is measured using a Kipp Solari sensor (Hortimax S.L., Almería, Spain), with a measurement range of 0 to 2000 W m^−2^, accuracy of ± 20 W m^−2^, and a resolution of 1 W m^−2^.

To determine the thermal difference (Δ*T*) that can be obtained through the evaporative cooling pad system, the fundamental indicator used to evaluate the operation benefits was the air saturation efficiency (*η*), which is defined as the quotient between the temperature decrease produced in the air when crossing the pad and the maximum possible decrease in air saturation conditions:

(1) $$\eta =\frac{T_{1}-T_{2}}{T_{1}-T_{wb}},$$

where *T_1_* is the dry temperature of the entering air (°C), *T_2_* is the dry temperature of the exiting air (°C), and *T_wb_* is the thermodynamic temperature of the wet bulb at entry.

If the saturation efficiency (*η*) of the pad, from the above equation, is known for a given air flow velocity, it is possible to determine the thermal difference (Δ*T*) produced by the pad by multiplying this efficiency by the wet-bulb depression (*WBD*), which is the difference between the dry entering air temperature (*T*_1_) and the wet-bulb temperature (*T_wb_*):

(2) $$\Delta T=T_{1}-T_{2}=\eta \cdot \left(T_{1}-T_{wb}\right),$$

where the temperature of the external wet bulb (*T_wb_*) is obtained from the dry temperature (*T_1_*), relative humidity, and atmospheric pressure of the external air [24].

We can also define the water consumption (*C_w_*) of the substrates (kg h^−1^ m^−2^ °C^−1^), expressed as the mass flow of evaporated water (*m_e_*) per unit of exposed surface (*A*) and the thermal difference (Δ*T*) that we can obtain for certain air conditions at the entry of the substrate.

(3) $$C_{w}=\frac{m_{e}}{\left(T_{1}-T_{2}\right)\cdot A},$$

where the flow of evaporated water (*m_e_*) is obtained by applying the water vapor balance:

(4) $$m_{e}=m_{v2}-m_{v1},$$

Dividing Equation (3) by the flow of dry air (*m_a_*) in kg h^−1^, which is constant between the entry and exit of the substrate and which we express as:

(5) $$m_{a}=\rho_{a}\cdot Q_{a},$$

where $\rho_{a}$ is the air density (kg m^−3^) and *Q_a_* is the air flow through the pad (m^3^ h^−1^), we obtain:

(6) $$m_{e}=m_{a}\cdot \left(W_{2}-W_{1}\right)=Q_{a}\cdot \rho_{a}\cdot \left(W_{2}-W_{1}\right),$$

where *W*_1_ and *W*_2_ are the absolute humidity of the air at the entry and exit of the pad, respectively (kg_w_ kg_a_^−1^).

Substituting the Equations (2) and (6) into Equation (3), the water consumption of the substrate depends on the airflow rate, saturation efficiency of the pad, and air conditions at the entry of the substrate, resulting in:

(7) $$C_{w}=\frac{Q_{a}\cdot \rho_{a}\cdot \left(W_{2}-W_{1}\right)}{\eta \cdot \left(T_{1}-T_{2}\right)\cdot A},$$

For the calculation of the above parameters, a set of expressions was used that is a result of the thermophysical properties of the air and of the vapor pressure of the water [25].

A saturation efficiency (*η*) and water consumption (*C_w_*) of 67.71% and 2.29 l h^−1^ °C^−1^ m^−2^, respectively, [26] for a flow speed recommended by the American Society of Agricultural and Biological Engineers (ASABE) of 1.27 m s^−1^ [27] are used for a 60–30° pad of 100 mm thickness manufactured by Munters (Kista, Sweden); this pad is widely used in Mediterranean greenhouses.

For this study, the period of time when evaporative cooling pads are usually used in the Almería region, from April to October, is considered due to the increase in outdoor temperatures, and in terms of the duration of operation, a total of 10 consecutive hours were considered from 10:00 to 19:00, when outdoor temperatures are higher.

### 2.2. Determination of Operating Parameters of Evaporative Control Pads

**Figure 1:**
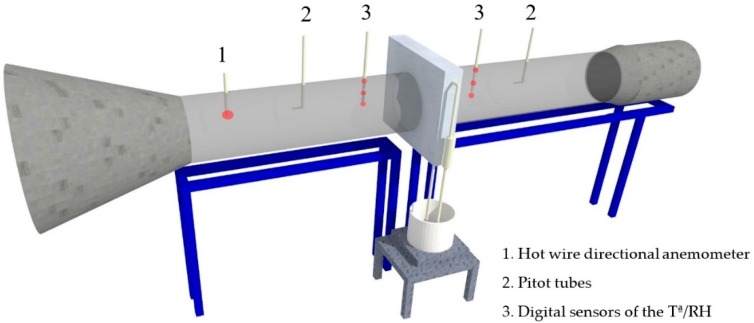
Schematic of wind tunnel used in tests with evaporation panels.

A low velocity wind tunnel designed and built at the University of Almería [28] was used (Figure 1) and adapted to test evaporative cooling pads [29], where the static pressures were measured in the test section using two 4 mm diameter Pitot tubes (Airflow Developments Lmt., Buckinghamshire, United Kingdom) placed 450 mm upstream and downstream of the central axis of the test section. The static pressure outputs of both tubes were connected to an SI 727 pressure transducer (Special Instruments, Nörlingen, Germany) that provides an output signal between 0 and 10 V with changes in the variable measured.

For the measurement of the air flow velocity, a hot wire directional anemometer EE70-VT32C5 (Elektronik, Engerwitzdort, Austria) with a measurement interval between 0 and 10 m s^−1^ and an accuracy of ± 0.1 s^−1^ was used, placed 950 mm before the test section. The maximum speed used in the tests was 3 m s^-1^ higher than twice the speed recommended by ASABE for corrugated cellulose pads [29].

The temperature and humidity of the air current were obtained through six digital sensors of relative humidity/temperature of the SHT75 series (Sensirion, Zurich, Switzerland), with a digital output of 9 bits, that did not need calibration, and with a relative humidity accuracy of ±1.8% and a temperature accuracy of ±0.3 °C. The sensors were located 700 mm upstream and downstream of the test section, mounted in groups of three on guides that were placed vertically along the diameter of the tunnel section.

The airflow was regulated by controlling the speed of the fan, and was monitored continuously using a hot wire anemometer. The air velocity during the test ranged between 0.3 and 4 m s^−1^. The tests began with a fixed flow of water. After 10 min the fan was started at an initial velocity of 0.3 m s^−1^, increasing up to 4 m s^−1^ in six steps and then decreasing once again to the initial velocity in order to check for possible errors of hysteresis in the sensors. Each increase and decrease in airflow was therefore approximately 0.6 m s^−1^, and after each variation the material was allowed a 5-min interval in which to readapt to the new conditions of air and water flow before measurements were taken. At each air velocity, 100 data points were recorded at approximately 3 s intervals by each of the sensors used.

The evaporative cooling pad analyzed in this study is installed in a multi-tunnel greenhouse belonging to a commercial nursery located in the municipality of La Mojonera (Almería) (36°48′6″ N, 2°41′14″ W), composed of 4 modules 26 m wide (4 × 6.5 m) and 43.7 m long. The greenhouse has 4 windows occupied by Munters brand evaporative cooling pads 60–30° 50 mm thick, with surfaces of 5.25 × 2 m, and 5 Munters EM50 fans located on the opposite side.

**Figure 2:**
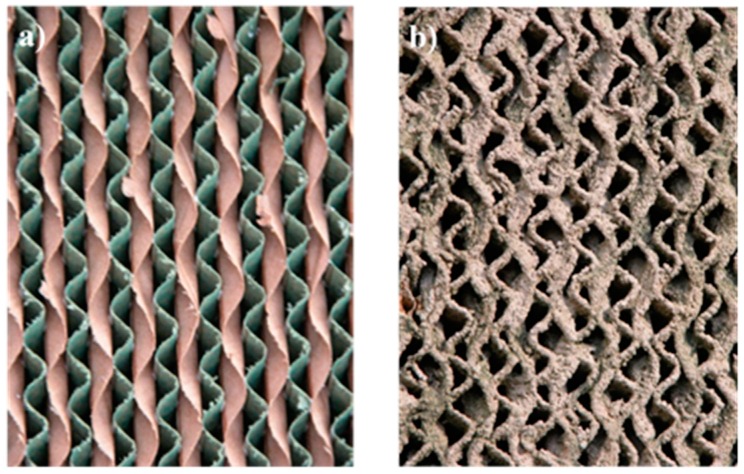
Munters 60–30° 50 mm cellulose evaporative cooling pads tested: (**a**) new and (**b**) after 3 years of operation.

The evaporative cooling pads are 3 years old and have copious incrustations of calcium carbonate precipitates (Figure 2). The precipitates alter the operation of the pads with respect to a new pad of the same model. This difference is why the pads were dismounted from one of the windows, and 3 samples 0.6 m wide by 0.65 m high were taken for analysis, and the pressure decrease and the saturation efficiency these pads produce with air flow in a wind tunnel were analyzed. These operating parameters were compared with those of a new pad of the same model and size to see how the parameters change over time.

## 3. Results

### 3.1. Thermal Difference and Mean Water Consumption Produced by the Pads

The mean temperature, relative humidity, and solar radiation of a historical series of 11 consecutive years (2008–2018) obtained by an outdoor weather station near the coastal zone of Almería in a period from 1 April to 31 October and from 10:00 to 19:00 are analyzed. The mean data of the three parameters are calculated by categorizing the data into three levels: maximum, mean and minimum.

**Figure 3:**
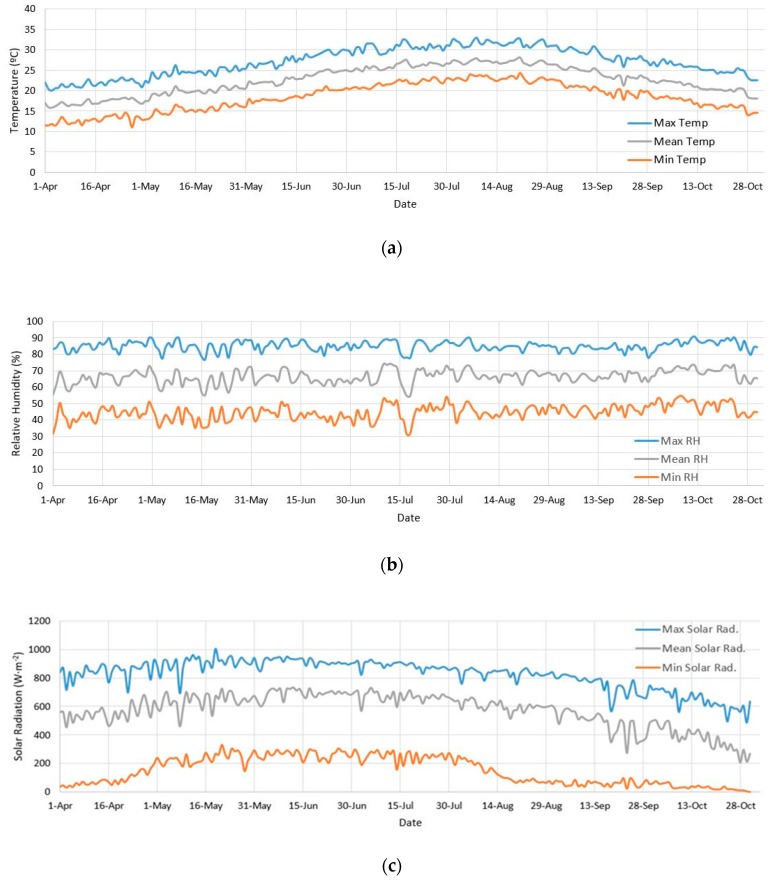
Mean evolution of the meteorological data (2008–2018) for: (**a**) temperature (°C), (**b**) relative humidity (%), and (**c**) solar radiation (W m^−2^).

The results obtained (Figure 3) show that the mean temperature in this period oscillates between 17.1 °C in April and 27.01 °C in August, with the maximum mean temperature for the same months being between 21.63 and 31.6 °C.

The mean relative humidity ranges from 63.46% in May to 69.91% in October. The minimum values for these months are between 40.56% and 49.27%, and the maximum values are between 84.33% and 87.42%, respectively. The mean solar radiation oscillates between 700.78 W m^−2^ in June and 370.17 W m^−2^ in October. The maximum values are found in June at 924.12 W m^−2^ and 51.55 W m^−2^ October.

**Figure 4:**
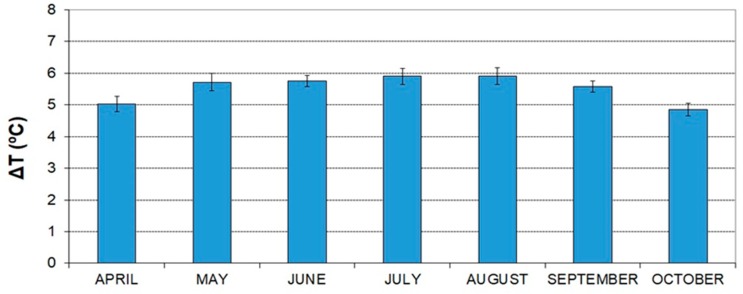
Evolution of the mean thermal difference (Δ*T*) produced by evaporative cooling pads in the coastal region of Almería.

For the mean values of temperature and external relative humidity, we can calculate the wet-bulb temperature with psychrometric software, and knowing the efficiency of the pad, we can apply Equation (2); we can determine the mean thermal difference of the evaporative cooling pads. These values are shown in Figure 4, obtaining mean values of 5.92 °C for the temperature reduction in the month of August and 4.85 °C for the month of October.

**Figure 5:**
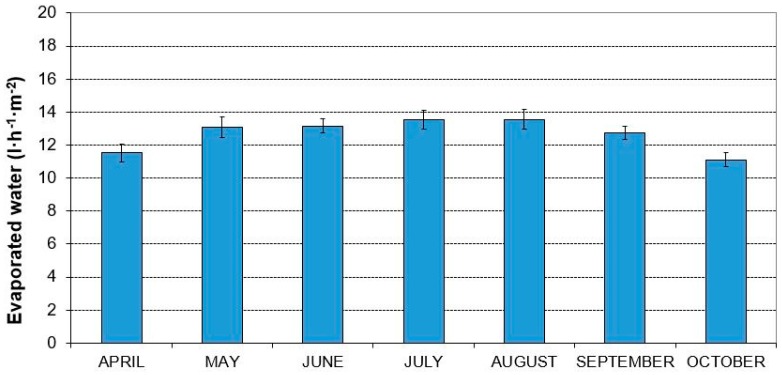
Evolution of the mean evaporated water (liters/hour) per unit area (m^2^) of the evaporative cooling pad in the coastal region of Almería.

Regarding the water consumption of the pads or the evaporated water per unit pad surface (L m^−2^ h^−1^), applying Equation (6), we obtain a mean result between 13.55 L m^−2^ h^−1^ in the month of August and 11.11 L m^−2^ h^−1^ in October (Figure 5).

### 3.2. Comparison of the Pressure Drop and Saturation Efficiency of the Evaporative Cooling Pad

**Figure 6:**
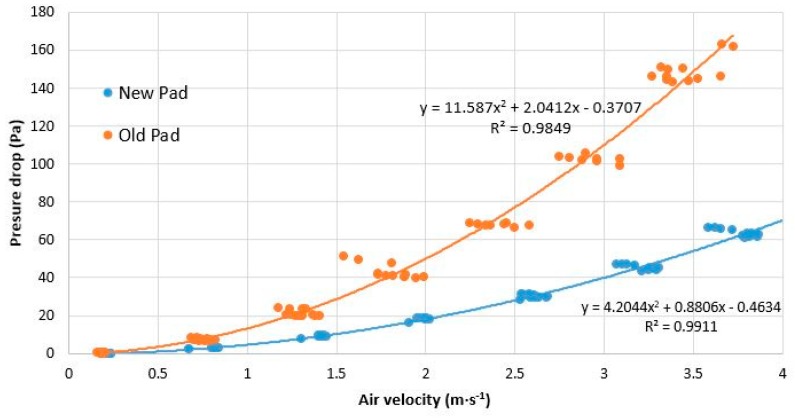
Comparison between the pressure decrease and air velocity produced by the 60–30° 50 mm pad used in the test greenhouse with respect to a new pad.

The pressure drop and saturation efficiency at different air velocities were determined in a low-speed wind tunnel. Both operating parameters were obtained in an evaporative pad installed in a greenhouse during 3 years of use and a new evaporative pad of the same geometry and model. As shown in Figure 6, the used pad has a greater pressure decrease than the new pad. This result is due to the decrease of the porosity of the pad from the incrustations of calcium carbonate on the surface of the cellulose. The greater the pressure decrease means that the fans have to exert more effort to get the air through the pads, reducing the flow rate and therefore the renewal rate. The mean increase in the pressure decrease compared with the unused pad (new) for a range of air flow velocity between 0.5 and 4 m s^−1^ is 179.04%, and within the recommended speed range between 1 and 1.5 m s^−1,^ the pressure drop increased between 8.59 and 18.36 Pa.

**Figure 7:**
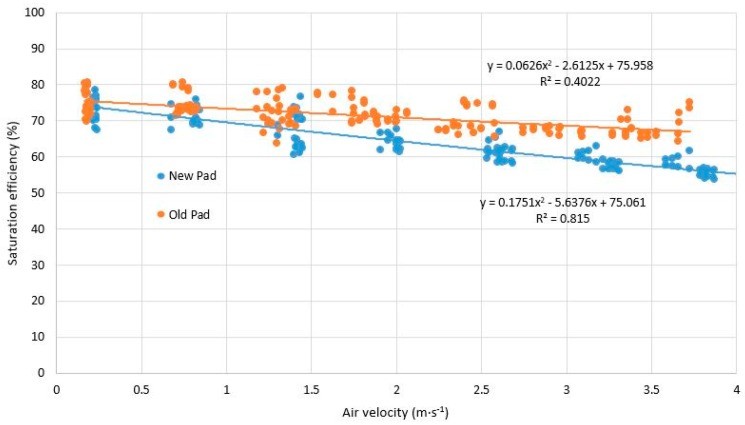
Comparison between the saturation efficiency and air velocity produced by the 60–30° 50 mm pad used in the test greenhouse with respect to a new pad.

The tests were done for the air saturation efficiency at different flow rates and water flow rates, and there was greater variability between the different samples, and counterintuitively, the saturation efficiency for any airflow rate for the used pad (old) was greater than that for the new pad (Figure 7). This result occurs because the used pad offers greater resistance to air flow, as seen in the previous section, and therefore, since it is harder for the air to cross the pads, the air is in contact with the water for longer, increasing the evaporation rate and producing a greater thermal difference. For the recommended flow rates (1–1.5 m s^−1^), the saturation efficiency of the used pad (old) ranges between 73.41% and 72.18%, a mean increase of 6.6% over that of the new pad.

## 4. Discussion

By analyzing the refrigeration capacity of the cellulose evaporative cooling pads, we find that this method is very effective in controlling the temperature and relative humidity inside a greenhouse, even in climatic zones with high humidity, as shown in other studies [13,14]. Despite the high humidity in the central hours of the day, we obtain mean reductions in air temperature of 5.92 °C in the month of August using the evaporative cooling pad. These values are very similar to those obtained in other studies in humid regions [12,30]. In addition, the mean evaporative cooling pad water consumption is 13.55 L m^−2^ h^−1^.

However, it is necessary to have a maintenance plan for evaporative cooling systems using cellulose pads and fans because the pressure decrease in an old pad increases by 179.04% with respect to that of a new pad due to a decrease in the porosity of the pad from incrustations on the pad, which decreases the air flow over time within the greenhouse. In contrast, the saturation efficiency increases by 6.6% due to the longer contact time between the water and the air that flows through the pad.

These results support the implementation of a new line of research that focuses on analyzing the variation over time and the use of the operating parameters of pad-fan systems installed in greenhouses.

## 5. Conclusions

According to the analysis of the operating parameters and cooling capacity of the evaporator panels, considering the historical climatic series of one of the areas with the highest concentration of greenhouses in the world (Almeria, Spain), we can conclude that evaporative cooling pads are applicable even in humid areas (coastal areas), provided that there is an adequate time variation of the thermohygrometric regime. Despite the high relative humidity of the air in the hottest hours of the day, a decrease of 5.92 °C in the mean temperature and a water consumption of 13.55 l/h per square meter of an evaporative cooling pad are obtained in the month of August.

The aging of the cellulose evaporator panels causes a drastic reduction in their porosity, increasing the necessary energy consumed by the system to maintain the setpoint ventilation rate. Over time and with low maintenance, the porosity of the pad decreases due to salt incrustation. The salt incrustation makes airflow more difficult in the pad, increasing the pressure drop by 170.04%; however, the air saturation efficiency of the pad increases by 6.6% due to the greater contact time between the air and the water.
